# Surveillance of Non-Malignant Asbestos-Related Diseases in an Exposed Population: A Scoping Review

**DOI:** 10.5334/aogh.4983

**Published:** 2026-02-19

**Authors:** Camilo de la Pava-Cortés, Esperanza Peña Torres, Tim Driscoll, Catherine Jones, Jennifer Coles, Shane McArdle, Kim Brislane, Matthew Peters, Guillermo Villamizar, Eduardo Algranti, Arthur Frank

**Affiliations:** 1 Department of Clinical Epidemiology and Public Health, Iberoamerican Cochrane Centre, Biomedical Research Institute Sant Pau (IIB Sant Pau), Barcelona, Spain; 2 Facultad de Enfermería, Pontificia Universidad Javeriana, Bogota, Colombia; 3 Asbestos Dust Diseases Research Institute (ADDRI), Sydney School of Public Health, University of Sydney, Australia; 4 Asbestos Dust Diseases Research Institute (ADDRI), University of Sydney, Australia; 5 Asbestos Dust Diseases Research Institute (ADDRI), Australia; 6 Asbestos Dust Diseases Research Institute (ADDRI) and Faculty of Medicine, Health and Human Sciences, Macquarie University, Australia; 7 Fundación Colombia Libre de Asbestos (FUNDCLAS), Bogotá, Colombia; 8 Fundação Jorge Duprat Figueiredo de Segurança e Medicina do Trabalho (FUNDACENTRO), São Paulo, Brazil; 9 Drexel University Dornsife School of Public Health, Philadelphia, PA, United States

**Keywords:** epidemiological monitoring, occupational health, public health surveillance, registry systems, toxic exposure

## Abstract

*Background:* Asbestos remains a significant global public health issue, with approximately 255,000 deaths attributed to exposure each year, primarily through occupational contact. Mesothelioma rates continue to rise, particularly in areas with a history of industrial exposure. Despite this burden, many countries lack reliable surveillance systems. Colombia has clusters like the one observed in Sibaté, highlighting the urgency of establishing structured, evidence-based surveillance systems.

*Objective:* The aim is to synthesize international experiences to guide the design and implementation of surveillance strategies in Colombia and other low- and middle-income countries facing similar challenges.

*Methods:* Following the JBI methodology for scoping reviews, comprehensive searches were conducted in Medline (PubMed), Embase, the Cochrane Library (OVID), and Google Scholar. Only English-language articles were included, and no time restrictions were applied.

*Results:* Fourteen studies from 11 countries were included, with the majority coming from Italy, followed by Colombia and Brazil. Three main themes emerged: (1) numerous cohort studies reported increased risks of mesothelioma and lung cancer among asbestos-exposed workers, supporting the need for long-term follow-up; (2) structured surveillance systems—such as Italy’s ReNaM and Brazil’s Datamianto—demonstrated effective models combining data integration, regular medical evaluations, and policy enforcement; (3) considerable variability in surveillance design, target populations, and reporting standards, especially between high-income and resource-limited settings, highlighting the lack of global standardization.

*Conclusions:* Structured, context-specific surveillance programs are essential to identify and manage the health burden of asbestos exposure. International models offer practical frameworks that could be adapted to Colombia’s needs. Investing in such systems would strengthen public health responses, improve early detection of asbestos-related diseases (ARDs), and support environmental and occupational justice in affected communities. The included studies do not mention monitoring according to the degree of exposure.

## Introduction

### Worldwide status

Asbestos remains a global health crisis, causing about 255,000 deaths annually, mainly from occupational exposure. All forms, including chrysotile, are known carcinogens linked to cancers like mesothelioma and lung cancer. Despite this, over 2 million tons are still used each year, mostly in lower income countries, with one death estimated per 20 tons consumed.

Asbestos consumption has been closely linked to the development of asbestos-related diseases (ARDs), including mesothelioma and asbestosis. Previous studies, such as the 2007 ecological study, identified a strong association between historical asbestos consumption and mortality from these diseases. A more recent analysis by Rath et al. [1] builds on this work, further confirming that higher asbestos consumption correlates with increased ARD mortality, particularly mesothelioma. Despite some progress in asbestos bans, many countries, especially in the developing world, continue to face significant health burdens due to asbestos exposure. This ongoing public health issue underscores the urgent need for stronger policies and global action to eliminate ARDs. Understanding the ecological relationships between asbestos exposure and health outcomes is critical for informing these policy efforts and advancing public health strategies [1]. Mesothelioma rates continue to rise, and economic losses in the EU alone account for 0.70% of GDP. Current safety limits are inadequate, prompting calls for stricter exposure caps, a total asbestos ban, and renewed global action from the ILO and WHO. Furuya et al. [2] provide a comprehensive global overview of the health impacts of asbestos exposure, emphasizing that the majority of attention in both historical and contemporary literature has been given to malignant diseases, particularly mesothelioma and lung cancer. While asbestosis, a non-malignant lung disease, was the first condition recognized as caused by asbestos, the scientific and regulatory focus has increasingly centered on its carcinogenic effects. The authors note that most estimates, statistics, and regulatory actions—such as those by WHO, ILO, and national health agencies—have concentrated on asbestos-related cancers, including mesothelioma, lung, laryngeal, and ovarian cancers. This bias in literature and health statistics may lead to an underappreciation of non-malignant diseases and the broader range of ARDs. The paper aims to consolidate recent global data on ARDs, with the primary focus on quantifying the burden of cancer. It also calls for the revitalization of international efforts to eliminate asbestos use and manage the risks of legacy exposure. Despite increasing evidence of the magnitude of the asbestos problem, especially cancer, policy responses remain insufficient in many parts of the world [2].

**Figure 1:**
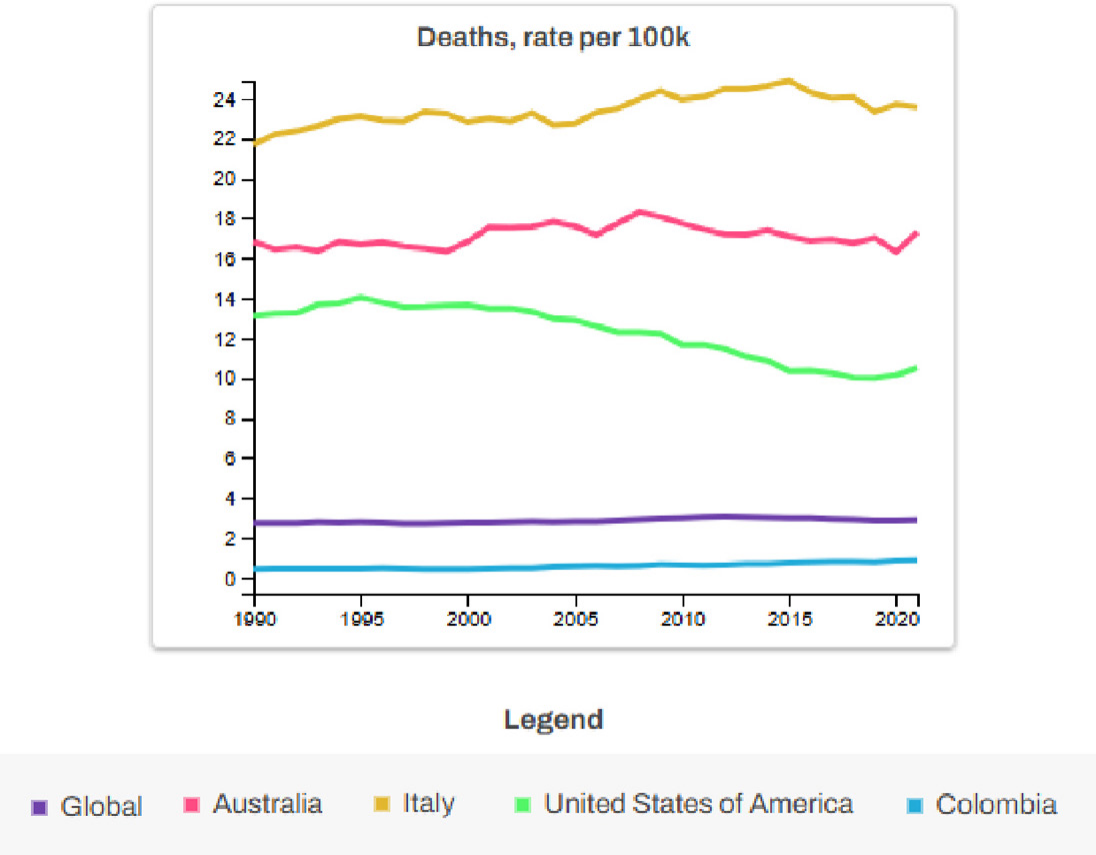
Deaths from occupational asbestos exposure, global and countries of interest, from 1990-2021 for both sexes and all ages IHME [3].

According to Figure 1, the country with the highest mortality was Italy, followed by Australia and the United States of America. The global trend was lower compared to the countries mentioned above. Colombia was below the global trend; however, this trend may be associated with under-registration, as concluded by Ramos-Bonilla et al. [4]. This study emphasizes the urgent need for Colombia to establish a reliable epidemiological surveillance system for ARDs. Active surveillance strategies can play a crucial role in identifying mesothelioma clusters and enhancing our understanding of the health effects of asbestos exposure in low- and middle-income countries (LMICs) [4]. A recent study by Moyano-Ariza et al. [5] further reinforces this point, highlighting that mesothelioma mortality in Colombia is heavily concentrated in urban areas with a history of asbestos exposure, such as Sibaté. The study also identifies a significant proportion of cases classified as “unspecified site,” underscoring the diagnostic limitations and the need for improved histopathological and diagnostic practices across the country. Despite recent progress, including the 2019 asbestos ban, ARDs continue to impact public health in Colombia due to underreporting and fragmented health data. The study calls for the establishment of a national mesothelioma registry and the strengthening of Colombia’s public health strategies to better address the long-term impacts of asbestos exposure. Such actions are crucial in reducing the disease burden and improving surveillance and care for affected populations.

A surveillance program is an ongoing scrutiny of a population (general population, study population, target population, etc.), generally using methods distinguished by their practicability, uniformity, and frequently their rapidity, rather than by complete accuracy [6].

### Colombia status

Since 1942, Colombia has hosted seven asbestos-processing plants, leaving behind asbestos-laden materials and exposing both workers and nearby communities to the fiber. A malignant pleural mesothelioma (MPM) cluster has also been identified in Sibaté—the site of the nation’s first fiber cement factory—primarily affecting local residents to date [4].

No comprehensive assessment has yet been done in other regions where asbestos plants operated to gauge potential spikes in related diseases. In our study, we estimate that about 10,000 people live within 500 m of a former plant or mine, while nearly 6 million—around 14% of Colombia’s population—reside within a 10-km radius. These numbers underscore the need for systematic surveillance of ARDs and environmental exposures in all areas around past asbestos facilities [7].

Accordingly, it is vital to implement health monitoring programs for current and former asbestos workers and their families, to identify any remaining sources releasing fibers into the environment, and to map and remediate sites contaminated by improperly disposed asbestos waste, especially near Colombia’s historical processing plants and mines [7].

Because of the lack of enforcement and surveillance of asbestos-processing facilities in LMICs, most studies have been conducted in high-income countries. The information available in the Colombian official morbidity and mortality database (SISPRO) was not reliable, since it did not identify the ARD cases that local inhabitants were denouncing [4].

## Review Question

What are the social and environmental conditions specific to a given setting that must be met for a collective epidemiological follow-up intervention to be provided to a community for probable risk of ARD?

Are the workers of the companies that used asbestos in Colombia for their production processes at a risk level lower than 5, eligible to enter the comprehensive health care pathway for people exposed to asbestos?

What should health surveillance for workers at risk of non-malignant disease due to asbestos exposure be like? What should its periodicity be?

## Inclusion Criteria

### Participants

Population exposed to asbestos.

### Concept

Exclusion criteria:

Studies that did not include a surveillance component;

Studies that included other minerals different from asbestos;

Studies focused on the legal aspects.

### Context

A worldwide approach to identify reported asbestos surveillance programs were reported, focusing on some insights, would be useful for the Colombian context to know the main characteristics and components of a surveillance program in different countries and contexts.

### Types of sources

Based on the experience of the working group, eligible sources included: primary studies (e.g., cohort, case-control, cross-sectional) that assessed surveillance programs related to asbestos exposure and its relation to non-malignant ARDs or exposure pathways and systematic reviews and meta-analyses, particularly those addressing surveillance programs related to asbestos. All types of studies, including systematic reviews, meta-analysis, and network meta-analyses, are discussed below.

## Methods

This scoping review was conducted following the JBI methodology for scoping reviews [8].

### Search strategy

MeSH and Emtree terms were described in Appendix I [6]. Free text terms were not included. Searches were conducted in Medline (PubMed), Embase, Cochrane Library, (OVID), and Google Scholar; the last resource was included to cover the gray literature. The search terms were reviewed by all members of the working group. Only articles in English were included. The search was carried out without a time frame, due to the body of evidence for the asbestos field arising from the 1980s.

### Study/source of evidence selection

For each source, a research information system file was downloaded, and afterward those files were uploaded to Rayyan [9] to perform the deduplication process using an automation process with a similarity threshold above 90%; the remaining references, as well as the screening phase, were resolved manually. This process was independently conducted by the two methodologists, and all conflicts were resolved without the involvement of a third reviewer. Details of this procedure and the reasons for exclusion of the full-text articles were reported in Appendix II PRISMA flow chart [10].

### Data extraction

Data extraction was carried out independently by two methodologists. Information was collected on an Excel file with the following fields: author, year, title, aim, study design, sample size, age, gender, country, manufacturing sector, surveillance program, characteristics of the surveillance program, results, and conclusion. These fields were discussed with the members of the working group. The details are presented in Appendix III, Data extraction.

### Data analysis and presentation

**Figure 2:**
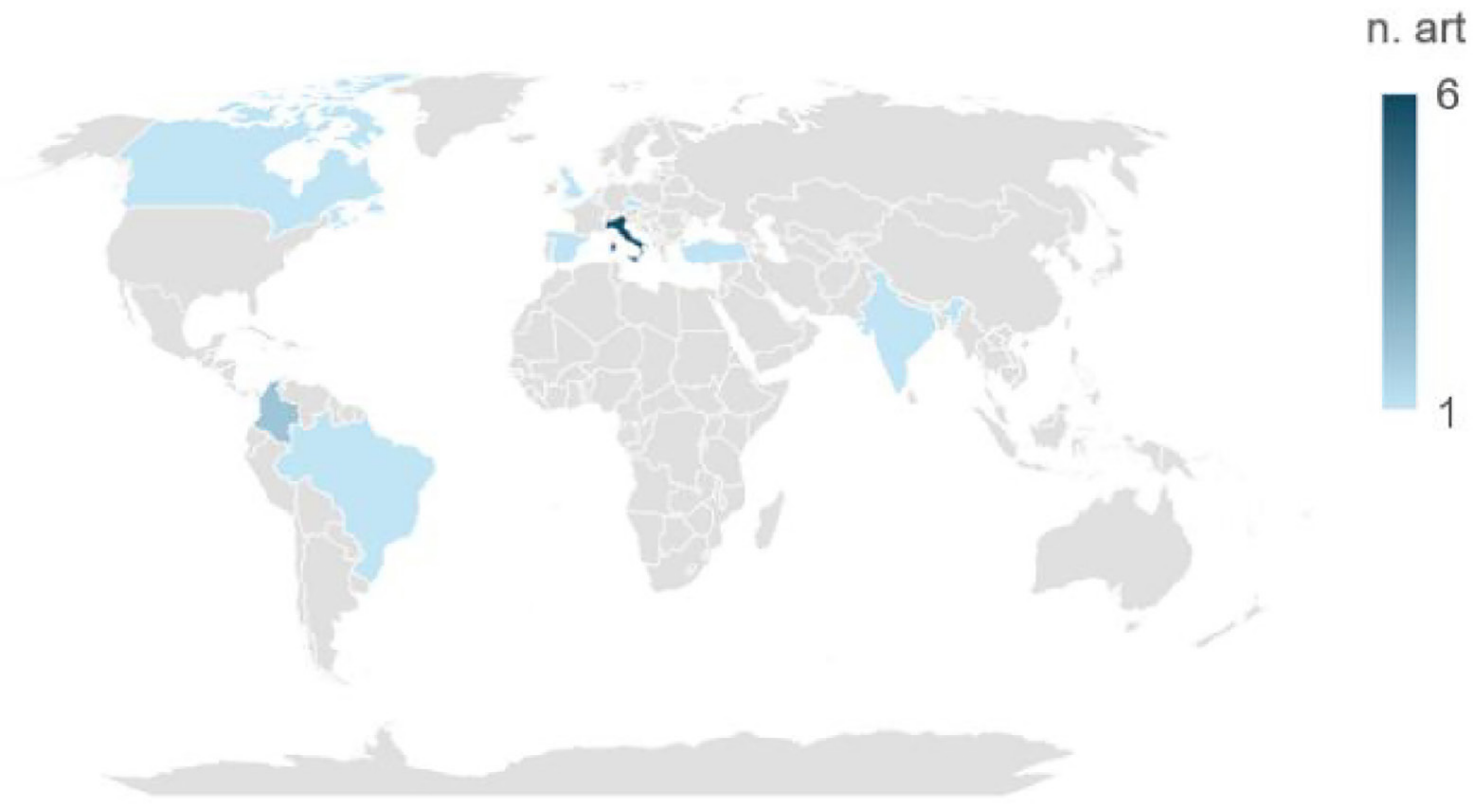
Worldwide distribution of analyzed articles. *The number of countries differs because one article was conducted across three countries. **Table:** 

A total of 14 articles were included, 6 of them were made in Italy, followed by 2 from Colombia and 1 for the rest of the included countries, the details are shown in Figure 2. The results of the scoping review can be summarized in three aspects:

1. *Increased risk in asbestos-exposed cohorts*
  Several studies document a clear excess in disease incidence and mortality among workers exposed to asbestos. Barbiero et al. [11, 12] reported two studies, with significantly elevated risks among 2488 male shipyard workers in Italy, and showed a standardized incidence ratio (SIR) of 8.82 for mesothelioma. Similarly, Comba et al. [13] reviewed the Casale Monferrato cohort and confirmed over 27,000 mesothelioma cases collected nationally through ReNaM between 1993 and 2015. Additionally, Chellini et al. [14] discussed the long-term health consequences among former asbestos-exposed workers, reinforcing the persistent impact of occupational exposure even decades after initial contact.
2. * Implementation of structured surveillance systems*
  A key finding across several studies is the establishment of formal monitoring systems for asbestos-related health risks. Buralli et al. [15] described the creation of “Datamianto,” a Brazilian surveillance platform, integrating software engineering with public health policy to track exposed populations and support healthcare planning. Comba et al. [13] detailed the Italian ReNaM system, a national registry focused on mesothelioma surveillance based on occupational and environmental exposure data. Similarly, Chellini et al. [14] provided insights into Tuscany’s public health organization, which developed a structured follow-up for exposed individuals, including clinical assessments and centralized data collection.
3. * Diversity in methodologies and program scope*
  The studies differ widely in research design, scope, and reporting completeness. Barbiero et al. [11] used historical cohort designs with detailed demographic and exposure data, while Buralli et al. [15] adopted a descriptive approach focusing on system development. In contrast, Chellini et al. [14] conducted a narrative study emphasizing program organization over epidemiological metrics. Furthermore, while the Italian studies provided detailed sex and age distributions, the Brazilian study lacked such demographic granularity. This variability underscores a broader challenge: the need for methodological standardization across asbestos surveillance initiatives to ensure comparability, improve data quality, and enhance evidence-based policy-making.

COUNTRY	N ARTICLES
Italy	6
Colombia	2
Brazil	1
Canada	1
Czech Republic	1
India	1
Belgium	1
Spain	1
Turkey	1
UK	1
**Total**	16*

## Discussion

International evidence underscores the urgent need to strengthen Colombia’s ARD surveillance. Structured programs like Italy’s ReNaM and Brazil’s Datamianto demonstrate how centralized registries and long-term follow-up enhance case detection and inform public health policy. These models offer valuable frameworks for adaptation in LMICs.

Environmental exposure, often underestimated, poses significant risks. Metintas et al. [16] show that prolonged contact with asbestos-contaminated environments can lead to lung cancer rates comparable to occupational exposure. In Colombia, areas like Sibaté exemplify this risk, yet national data systems remain fragmented and underreport non-malignant conditions.

Surveillance strategies must go beyond occupational cohorts. Merler et al. [17] emphasize that medical monitoring should extend past employment due to the long latency of ARDs. Their findings also highlight the limited effectiveness of chemoprevention trials, reinforcing the need for early detection and tailored screening protocols.

Exposure quantification is another critical gap. Ramada Rodilla et al. [18] show that amphibole fibers ≥5 µm are most strongly associated with mesothelioma and lung cancer, while exposures below 0.1 f/ml are unlikely to cause malignancy. Colombia lacks reliable exposure data, making it difficult to align with international toxicological benchmarks.

To address this, Colombia should incorporate fiber-specific thresholds and cumulative exposure metrics into its surveillance systems. Integrating geospatial mapping, clinical follow-up, and centralized registries—modeled after ReNaM and Datamianto—would bridge the gap between global standards and local realities.

The systematic review by Santos et al. [19] further reinforces the link between asbestos and MPM, with a latency period of around 42 years and near-total mortality. Their call for national mesothelioma surveillance centers aligns with Colombia’s need for improved diagnostic infrastructure and registry development.

Together, these studies advocate for coordinated global and national action. Colombia must adapt proven international models to its context, ensuring that surveillance systems are inclusive, evidence-based, and responsive to both occupational and environmental exposures. This approach will advance public health equity and environmental justice for affected communities.

## Declarations

The members of the working group and their institutions are internationally recognized leaders in the field of asbestos research.

## Additional File

The additional file for this article can be found as follows:

Supplementary Material 1. Appendices.
