# Supplementary material for: Surveillance of Non-Malignant Asbestos-Related Diseases in an Exposed Population: A Scoping Review
Source: Ann Glob Health. 2026 Feb 19;92(1):19. doi: 10.5334/aogh.4983 (PMC12922659; doi:10.5334/aogh.4983)
Supplement: Supplementary Material 1. — Appendices. [file agh-92-1-4983-s1.pdf]

Supplementary Material

Appendix I: Search strategy

Category	MESH terms	Emtree Terms	Free text terms
Population	Asbestos	Asbestos	None
Intervention	Public Health Surveillance	Public Health Surveillance	
	Environmental Monitoring	Environmental Monitoring	
	Watchful Waiting	Watchful Waiting	
	Chronic Disease Indicators	Chronic Disease Indicators	
	Epidemiological Monitoring	Epidemiological Monitoring	

Search in Medline – Pubmed	
Database	Medline
Platform	Pubmed
Date of search	22-Mar-2025
Publication date	Without dataframe
Language filters	English
Other filters	None
Query	#1 "Asbestos"[MeSH Terms] OR "Asbestos"[Title/Abstract]<br>Hits: 15,957<br><br>#2 ((((((((Public Health Surveillance[MeSH Terms]) OR (Public Health Surveillance[Title/Abstract])) OR (Environmental Monitoring[MeSH Terms])) OR (Environmental Monitoring[Title/Abstract])) OR (Watchful Waiting[MeSH Terms])) OR (Watchful Waiting[Title/Abstract])) OR (Chronic Disease Indicators[MeSH Terms])) OR (Chronic Disease Indicators[Title/Abstract])) OR (Epidemiological Monitoring[MeSH Terms])) OR (Epidemiological Monitoring[Title/Abstract])<br>Hits: 217,413<br><br>#1 AND #2<br>Hits: 471
Results	471

Search in Embase	
Database	Embase
Platform	Embase
Date of search	22-Mar-2025
Publication date	Without dataframe
Language filters	English
Other filters	None
Query	#1 'asbestos'/exp OR 'asbestos':ti,ab,kw<br>Hits: 23,329<br><br>#2 'public health surveillance'/exp OR 'public health surveillance':ti,ab,kw OR 'environmental monitoring'/exp OR 'environmental monitoring':ti,ab,kw OR 'watchful waiting'/exp OR 'watchful waiting':ti,ab,kw OR 'chronic disease

Search in Embase	
	indicator'/exp OR 'chronic disease indicators':ti,ab,kw OR 'epidemiological monitoring'/exp OR 'epidemiological monitoring':ti,ab,kw<br>Hits: 133,349<br><br>#1 AND #2<br>Hits:391
Results	391

Search in Cochrane Library	
Database	Cochrane Library
Platform	OVID
Date of search	22-Mar-2025
Publication date	Without dataframe
Language filters	English
Other filters	None
Query	#1 "Asbestos".ab,sh,ti.<br>Hits: 182<br><br>#2 (Public Health Surveillance or Environmental Monitoring or Watchful Waiting or Chronic Disease Indicators or Epidemiological Monitoring).ab,sh,ti.<br>Hits: 1,604<br><br>#1 AND #2<br>Hits: 1
Results	1

Search in Google Scholar	
Database	Google Scholar
Platform	Google Scholar
Date of search	22-Mar-2025
Publication date	Without dataframe
Language filters	English
Other filters	None
Query	((“Asbestos”) AND (“Public Health Surveillance” OR “Environmental Monitoring” OR “Watchful Waiting” OR “Chronic Disease Indicators” OR “Epidemiological Monitoring”))<br>Hits: 60 out of 10,900
Results	60

Appendix II: PRISMA flow chart

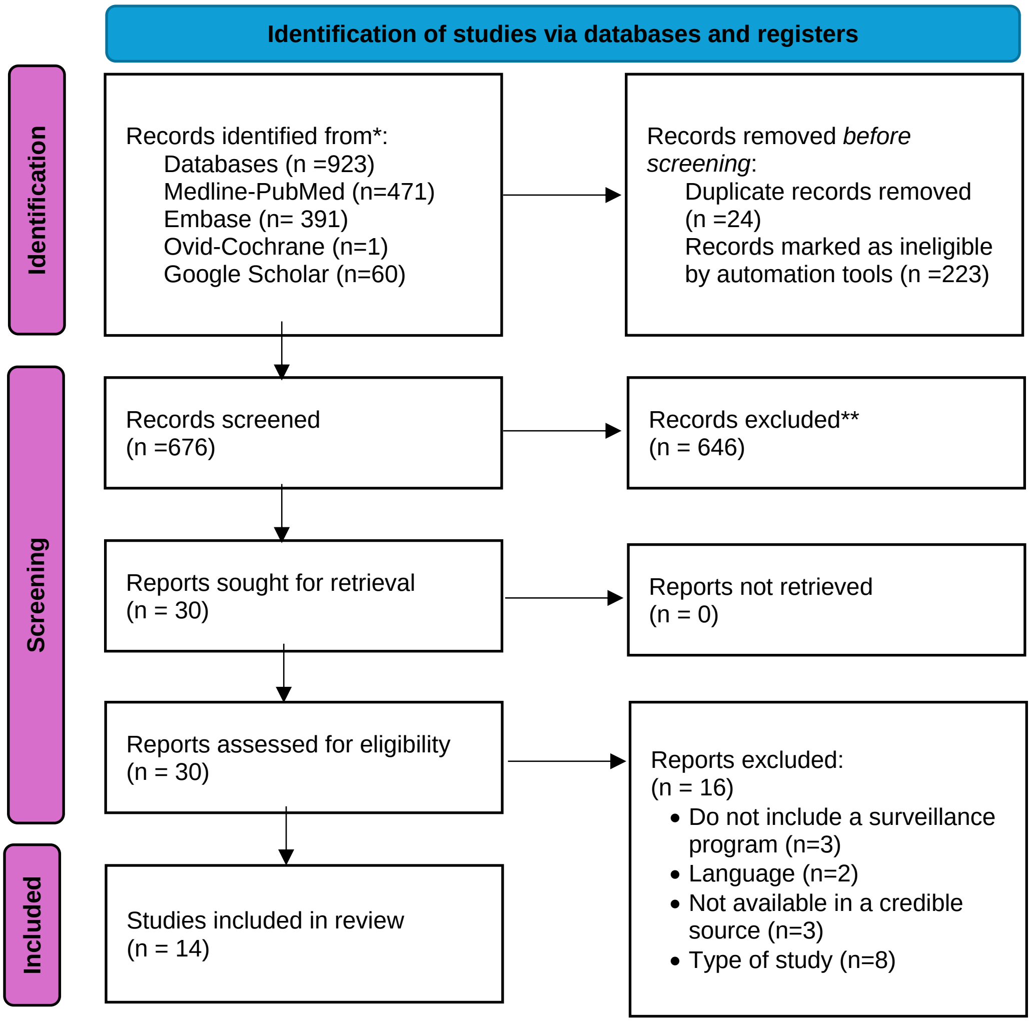

Source: Page MJ, et al. BMJ 2021 (10)

Appendix III: Data extraction

Author	Year	Title	Aim	Study design	Sample size	Age Years (SD)	Gender
Barbiero, F. et al (11)	2018	Cancer incidence in a cohort of asbestos-exposed workers undergoing health surveillance	To compare a local cohort of 2488 men occupationally exposed to asbestos and enrolled in a public health surveillance program with the 1995–2009 cancer incidence of the general population of Friuli Venezia Giulia (FVG) region, Northeast Italy.	Historical cohort study	2488	56.76 (11.99)	Men 100% Women 0%
Barbiero, F. et al (15)	2018	Mortality in a cohort of asbestos-exposed workers undergoing health surveillance	To compare mortality in a cohort of 2,488 men occupationally exposed to asbestos, enrolled in a PHSP in FVG between the early 1990s and 2008, with that of the general population of FVG and Italy.	Cohort study	2488	56.76 (11.99)	Men 100% Women 0%
Buralli, RJ. et al (14)	2023	The Brazilian system for monitoring workers and general population exposed to asbestos: development, challenges, and opportunities for workers' health …	To describe the development of a monitoring system for workers and the general population exposed to asbestos in Brazil and to discuss the main challenges and opportunities for worker health surveillance.	Descriptive study	3528	Not reported	Not reported
Chellini, E. et al (13)	2018	Health surveillance for former asbestos exposed worker: a specific programme developed in an Italian region	To describe the main clinical and organisational features of the regional programme of post occupational health surveillance for former asbestos workers.	Narrative study	Not reported	Not reported	Not reported
Comba, P. et al (12)	2018	Mesothelioma in Italy: the Casale Monferrato model to a national epidemiological surveillance system	To review the origin and development of the epidemiology of mesothelioma in Italy, starting with the detection and investigation of the major outbreak of the disease observed in Casale Monferrato, Piedmont Region.	Descriptive study	3434 workers at 2003 of which 777 women, that were followed-up until 2003.	Not reported	Men 2657 (77,4%) Women 777 (22.6%)
Jadhav, AV. et al (16)	2025	Understanding exposure risk using soil testing and GIS around an abandoned asbestos mine.	To detect the presence of asbestos in soil samples from residential areas and to understand its spread in the villages around the Roro mine over the years.	Experimental study	Not applicable	Not applicable	Not applicable

Jones, AD. et al (17)	2005	A comparison of fibre counting across three European national proficiency testing schemes.	To compare the target counting levels being set in three national PT schemes for asbestos fibre counting, by circulating 40 reference slides, drawn from the three schemes, to six laboratories (two from each national scheme).	Descriptive study	Not applicable	Not applicable	Not applicable
Klán, M. et al (18)	2018	New comprehensive approach for airborne asbestos characterisation and monitoring.	To determine the asbestos fibre sources in this urban air.	Experimental study	Not applicable	Not applicable	Not applicable
Lysaniuk, B. et al (7)	2021	Using GIS to Estimate Population at Risk Because of Residence Proximity to Asbestos Processing Facilities in Colombia.	To estimate the number of people from the general population living in distance bands from asbestos processing facilities and the asbestos mine at which an elevated risk of asbestos-related diseases (ARD) has been reported in the scientific literature.	Systematic review + geographical study	Not applicable	Not applicable	Not applicable
Mangone, L. et al (19)	2017	The effects of improving the mesothelioma surveillance network on sensitivity, timeliness in reporting and asbestos exposure assessment.	To evaluate the impact of the extension of the network on MR sensitivity and timeliness.	Descriptive study	2019	Age group = n - % <40 = 21 - 1.0 40-49 = 62 - 2.9 50-59 = 223 - 10.4 60-69 = 598 - 28.0 70+ = 1230 - 57.6	Men 1455 (72,0%) Women 564 (28,0%)
Metintaş, S. et al (20)	2017	Turkey National Mesothelioma Surveillance and Environmental Asbestos Exposure Control Program.	to determine the incidence of mesothelioma in Turkey, to identify asbestos exposed villages and MM incidence in the rural side, and to create a risk map for environmental asbestos exposure in Turkey.	Cohort - From case to the field	5617	Age group (%) <40 323 (5.8) 40–49 694 (12.4) 50–59	Male 3241 (57,7%) Female 2376 (42,3%)

						1362 (24.2) 60–69 1508 (26.8) 70+ 1730 (30.8)	
Pefoyo, AJK. et al(21) (22)	2014	Exploring the usefulness of occupational exposure registries for surveillance: the case of the Ontario Asbestos Workers Registry (1986–2012)	to evaluate the validity and usefulness of the data contained in the OAWR.	Cohort study	33011	workers aged between 15 and 80 years	Not reported
Ramos, JP. et al (21)	2023	An Approach to Overcome the Limitations of Surveillance of Asbestos Related Diseases in Low-and Middle-Income Countries: What We Learned	to provide a practical guide to investigators engaged in bringing to light previously undetected, if not undetectable, localized excesses of ARD whose discovery might open the way to environmental cleanup, health protection, and increased awareness of asbestos burden of diseases in the affected communities.	Surveillance study	17 cases	Not reported	Not reported
Scarlata, S. et al (23)	2017	Chest ultrasonography in health surveillance of asbestos related pleural disease.	To validate thoracic US as a diagnostic tool in the management of pleural changes in subjects with a history of occupational exposure to asbestos and to compare its diagnostic accuracy with that of HRCT scan of the chest.	Cross-sectional	117	46.8 years (± 6.0)	Male 100%
Proficiency testing (PT), E-R MR Emilia Rogmagna Mesothelioma Registry, MM Malignant Mesothelioma, OAWR Ontario Asbestos Workers Registry							

Continuation of the Data extraction

Author	Year	Country	Manufacturing Sector	Surveillance program	Characteristics of the surveillance program	Results	Conclusion
Barbiero, F. et al (11)	2018	Italy	Shipbuilding activities	Yes	- Demographics (name, surname, date of birth, residence and phone number); company name and industrial sector; total years of exposure to asbestos; cigarette smoking habits (never, ex or current smoker); type, date and findings of examination<br>- Cancer Registry follows cancer	Among cohort members the SIR was 8.82 (95% CI 5.95–12.61) for mesothelioma and 1.61 (95% CI 1.26–2.04) for lung cancer. In subgroup analyses, the SIR for lung cancer in subjects hired in shipbuilding between 1974 and 1984 was 2.09 (95% CI 1.32–	This cohort experienced an excess in the incidence of both mesothelioma and lung cancer, showing increasing incidence rates at higher level of asbestos exposure. For lung cancer, the relative incidence was highest among workers hired in

Author	Year	Country	Manufacturing Sector	Surveillance program	Characteristics of the surveillance program	Results	Conclusion
					registration rules according to WHO - IARC<br>- Following computerized data sources: Hospital Admissions, Pathology Reports, Death Records and Residential Status Records.	3.13). In the overall cohort, a borderline increased incidence was also found for stomach cancer (SIR = 1.53 95% CI 0.96–2.31). Internal comparisons within the cohort show that among men with high asbestos exposure level the relative risk was almost threefold for lung cancer (IRR = 2.94 95% CI 1.01–8.57).	shipbuilding between 1974 and 1984.
Barbiero, F. et al(15)	2018	Italy	Shipbuilding activities	Yes	- Demographics (name, surname, date of birth, residence and phone number); company name and industrial sector; total years of exposure to asbestos; cigarette smoking habits (never, ex or current smoker); type, date and findings of examination<br>- Cancer Registry follows cancer registration rules according to WHO<br>- IARC<br>- Death	A strong excess in mortality for PC with reference to FVG (SMR=6.87, 95% CI 4.45-10.17) and Italian population (SMR=13.95, 95% CI 9.02-20.64) was observed. For LC, the FVG-based SMR was 1.49 (95% CI 1.17-1.89) and the Italy-based 1.43 (95% CI 1.12-1.81).	This cohort experienced an excess mortality for pleural and lung cancer, compared with regional and national populations. For lung cancer, the excess was stronger in workers with the first hire in shipbuilding before 1985, suggesting a key role of asbestos exposure.
Buralli, RJ. et al (14)	2023	Brazil	Asbestos-related companies	Yes	Datamianto Surveillance: health promotion and disease prevention activities, considering workers' health and safety guidelines, NR-15 regulations, and health surveillance related to companies' obligations. Assistance: specialized health services related to the diagnosis of ARD, clinical followup, and treatment realized by primary, secondary, and tertiary care, which includes conducting periodic CXR, interpreted according to ILO Classification of Pneumoconiosis Radiographs, and spirometry.	The system was developed by a group of software developers, workers' health specialists, and practitioners, and it was recently incorporated by the Ministry of Health to be used for workers' health surveillance. It can facilitate the monitoring of exposed individuals, epidemiological data analysis, promote cooperation between health services, and ensure periodical medical screening guaranteed to workers by labor legislation. Moreover, the system has a Business Intelligence (BI) platform to analyze epidemiologic data	Datamianto can support and qualify the healthcare and surveillance of asbestos-exposed workers and ARD, promoting a better quality of life for workers and improving companies' compliance with legislation. Even so, the system's significance, applicability, and longevity will depend on the efforts aimed at its implementation and improvement.

Author	Year	Cou ntry	Manufacturing Sector	Surveillance program	Characteristics of the surveillance program	Results	Conclusion
						and produce near real-time reports.	
Chellini, E. et al (13)	2018	Italy	Former asbestos workers	Yes	First phase<br>- Collects his/her physiological anamnesis, medical history, and evaluates possible signs and symptoms of asbestos-related diseases; Prescribes a chest radiograph and its interpretation in accordance with the International Labour Organization (ILO) system for classification of pneumoconiosis<br>- Prescribes a spirometry including flow-volume curves<br>- Administers the European Coal and Steel Community (ECSC or CECA) questionnaire for the diagnosis of chronic bronchitis and the Medical Research Council (MRC) questionnaire for the evaluation of dyspnoea seriousness.<br><br>Second phase<br>Examination is applied, if needed, only for more in-depth evaluation of the health status of the subject in order to confirm doubts concerning the diagnosis of an asbestos-related disease, in cooperation with other health services/professionals (radiologists, pulmonologists and others). In particular, computed tomography (CT) examination is performed in order to evaluate pleural plaques and parenchymal abnormalities according to the ICOERD.<br><br>In order to guarantee a homogeneous health surveillance in the whole region, the Tuscan programme includes: Operational plans, one for each area (North-	In Tuscany, an organization of public health services devoted to this surveillance was defined, based on the national recommendations, characterized in terms of efficacy, suitability, social utility and economical sustainability. All these characteristics allowed to offer the programme free of charge within the regional public health services with the involvement of preventive (OHSs, antismoking services) and care services (unit of radiology and unit of lung diseases).	the identification of a specific public health surveillance programme for former asbestos workers including training and monitoring activities and the cooperation of professional and social stakeholders might facilitate to overcome still open problems as the lack of a diffuse knowledge of the service with a broaden invitation to adhere to the programme, the correct stratification of subjects for the follow-up and the real homogeneous delivery of the health surveillance in the whole region.

Author	Year	Country	Manufacturing Sector	Surveillance program	Characteristics of the surveillance program	Results	Conclusion
					West, Centre and South-East) where a local health administration has to organize and manage the specific health services for the programme; A continuous training of the involved health professionals; A regional technical coordination group; An evaluation and monitoring plan of the performed activities; A cooperation agreement with unions and association of former exposed workers.		
Comba, P. et al (12)	2018	Italy	Occupational and residential exposure to asbestos in the area of Casale Monferrato (Italy)	Yes	ReNaM is a national surveillance system of mesothelioma incidence, active with force of law since 2002, devoted to identify cases and to assess asbestos exposure modalities. An Operating Centre in all 20 Italian regions (COR, Centro Operativo Regionale), works applying standardized methods, as described in the national Guidelines [46]. CORs retrieve information about incident malignant mesothelioma (MM) cases from health care institutions potentially involved in diagnosis (chest surgery wards, pathology and lung care units) and classify the reliability of diagnoses according to 3 classes of decreasing level of certainty: certain (if histological confirmation is available), probable (if cytological confirmation is available) and possible MM (only radiological and clinical evidences)	Between 1993 and 2015, ReNaM has collected 27356 incident MM cases. Certain MM represent around 80% of detected cases. Incident case list for 2015 is ongoing. More than 90% of collected cases are localized in the pleura (93%), peritoneal MM cases are 6.5% (5.3% and 9.4% in men and women respectively) and pericardial and tunica vaginalis testis MM cases are very rare (58 and 79 collected cases respectively among the entire ReNaM archives).	The experience of Casale Monferrato represents a lesson in several terms, from the epidemiological surveillance to the health care of the victims and the relationship between epidemiologists, victims, their relatives and residents in contaminated areas. The studies performed in Casale have contributed to the implementation and interpretation of mesothelioma registration, temporal and spatial analyses of mesothelioma occurrence, analytical epidemiological investigations, health care planning, support to asbestos victims and community empowerment throughout all of Italy.
Jadhav, AV. et al (16)	2025	India	Mills and factories that process asbestos	No	Not applicable	The soil sample testing indicated that, out of 16 soil samples from residential areas, 12 showed the presence of chrysotile asbestos. It was found in the map analysis	The evidence indicated the presence of asbestos in the soil of nearby residential areas around the mine, and this contamination has spread over the years.

Author	Year	Country	Manufacturing Sector	Surveillance program	Characteristics of the surveillance program	Results	Conclusion
						that asbestos-containing areas had enlarged by around 20% in those years.	Similar studies at other mine locations are needed, and timely interventions are warranted to protect nearby residents.
Jones, AD. et al (17)	2005	Spain, Belgium and UK	UK - either asbestos removal or clearance testing and textile industry Belgium - maintenance work on materials containing asbestos and similar activities Spain - asbestos cement dust.	No	Not applicable	the UK and Spanish samples have national scheme reference values based on ERM counts, those national scheme reference values (R) are compared to the ERM counts made in the exchange. The Belgian scheme national reference values are based on WHO counts and therefore are compared to densities from the WHO counts in this exchange.	Since it is important to be able to compare fibrecounting measurements internationally, it is important to have consistency in the target levels being set by fibre-counting PT schemes. The consistency between the three schemes (in this study) would be a good platform for further comparisons to assess and develop comparability with other national PT schemes, perhaps as either special exercises such as this or within the process of regular international exchanges such as those in the AFRICA scheme.
Klán, M. et al (18)	2018	Czech Republic	construction aggregates for gravel production	No	Not applicable	Actinolite was identified in 40% of the PM samples. The relationship between the meteorology and presence of actinolite in the 24 PM10 samples was not proven, probably due to the long sampling integration time. Therefore, highly time-and-size-resolved PM sampling was performed. Second, sampling of size-segregated aerosols and measurements of the wind speed (WS), wind direction (WD), precipitation (P)	In spite of numerous areas of airborne asbestos fibres all over the world, asbestos monitoring relies solely on morphology/mineralogy and/or the chemical composition of asbestos fibres in bulk samples and PM.

Author	Year	Country	Manufacturing Sector	Surveillance program	Characteristics of the surveillance program	Results	Conclusion
						and hourly PM10, PM2.5 and PM1 were conducted in a suburban locality near the quarry in two monthly highly time-resolved periods (30, 60, 120 min).	
Lysaniuk, B. et al (7)	2021	Colombia	Asbestos cement and Friction products	No	Not applicable	The results of the current study reaffirm the importance of implementing mesothelioma registries and other surveillance strategies for asbestos-related diseases in Colombia, especially in the regions where asbestos processing plants or the mine are located, and the urgent need to develop and implement a National Plan for the Elimination of Asbestos-related Diseases.	In this study, we estimated the number of people living in Colombia in close proximity to asbestos processing facilities and the only asbestos mine in the country (up to a distance of 10 km). Within these distances, studies conducted in other parts of the world have found an increased risk of ARD, especially mesothelioma.
Mangone, L. et al(19)	2017	Italy	Asbestos-related industries	Yes	To assess timeliness reached by surveillance system, we measured median times (in days) by certainty level (certain MM or not) comparing diagnosis/ reporting to E-R MR dates and diagnosis/interview dates. Date of reporting coincided with the first notification, made by one network member or by a completion source (hospital discharge records or mortality records). We reported also asbestos exposure according to interview responder (patients or their relatives).	Sensitivity increased from 79.4% (1996-2001), to 89.0% (2002-2007) and to 91.4% (2008-2013). For mesothelioma with diagnostic certainty, we recorded considerably reduced reporting times from the 50th percentile on, whereas for uncertain mesothelioma relevant reductions were observed also in the lower percentiles. A reduced time to interview was observed too, which was more significant for uncertain cases. The proportion of patients directly interviewed increased from 33.5% (1996-	The extended network improved the MR sensitivity and allowed shorter reporting and interview times and more frequent patient interviews, thus improving accuracy of exposure definition.

Author	Year	Country	Manufacturing Sector	Surveillance program	Characteristics of the surveillance program	Results	Conclusion
						2001), to 39.1% (2002-2007), to 49.5% (2008-2014).	
Metintaş, S. et al (20)	2017	Turkey	Villages with Asbestos Exposure	Yes	Turkey National Mesothelioma Surveillance and Environmental Asbestos Exposure Control Program (TUNMES-EAECP): Registry, village surveillance, geographical analysis, soil samples.	The number of confirmed MM cases was 5617 with a male to female ratio of 1.36. Mean age was 61.7 13.4 (20–96) years. The median survival was eight (95% CI 7.6–8.4) months. Asbestos exposure continues in 379 villages, with 158,068 people still living in high risk areas.	In conclusion, asbestos exposure in rural areas remains a critical problem in Turkey. This study is the first time that the size of the problem and risk of MM was comprehensively identified on a nationwide basis for Turkey. In addition, people at risk were also determined.
Pefoyo, AJK. et al (22)	2014	Canada	Construction and others sectors	Yes	Registry, follow-up and measure asbestos-exposure	The incidence of exposure started to decrease around 1990; but about 2000 workers were still exposed annually until 2006. Results showed large geographical disparities. Unexpectedly, workers from industries other than construction reported exposure.	The Ontario Asbestos Workers Registry is a useful but challenging source of information for the surveillance of asbestos exposure in Ontario. The registry could benefit from well-defined surveillance objectives, a clear exposure definition, systematic enforcement, regular data analyses, and results dissemination.
Ramos, JP. et al(21)	2023	Colombia	Asbestos-related industries	Yes	2. reviewing existing sources of information that could be used to identify ard cases<br>3. finding the cases directly in sibaté<br>4. validation of the cases<br>5. estimation of the age-adjusted incidence rate for mesothelioma<br>6. determining potential exposure sources to asbestos for the cases<br>7. communicate with health and environmental authorities and the affected resident population the study findings and disseminate key messages to the public	The active surveillance strategy successfully identified a mesothelioma cluster in Sibaté, revealing the inadequacy of the existing health information system in monitoring asbestos-related diseases. The discovery of this cluster underscores the critical importance of implementing active surveillance strategies in Colombia, where governmental institutions and resources are often limited.	Conclusion: The findings of this study emphasize the urgent need for Colombia to establish a reliable epidemiological surveillance system for asbestos-related diseases (ARDs). Active surveillance strategies can play a crucial role in identifying mesothelioma clusters and enhancing our understanding of the health effects of asbestos exposure in

Author	Year	Country	Manufacturing Sector	Surveillance program	Characteristics of the surveillance program	Results	Conclusion
							low- and middle-income countries.
Scarlata, S. et al (23)	2017	Italy	aircraft and automotive maintenance operator, pilots, and on-board system operators	No	Not applicable	Thirteen out of 19 subjects with pleural abnormalities at HRCT were also identified by thoracic US, whereas 47 participants had lesions seen at US, but not at the HRCT scan. Positive and negative percent agreement was 66.6% and 51.8%, respectively; the McNemar's test for equality showed a p-value<0.001.	In conclusion, chest US might complement HRCT in the heath surveillance in asbestos exposed populations either to detect earlier lesions or to follow up US approachable lesions.
